# Transactions of the Philadelphia College of Physicians

**Published:** 1848-05

**Authors:** 


### TRANSACTIONS OF THE PHILADELPHIA COLLEGE OF PHYSICIANS.

The Summary of the Transactions of the College of Physicians of Philadelphia from December 1847, to March 1848, inclusive, has been published, and contains reports and communications on a great variety of topics of professional interest. The labors of this body of physicians have the proper direction, and the fruits are creditable to the profession of our country. We shall make room at an early day for extracts from this volume of their Transactions.

